# Intranasal deferoxamine attenuates synapse loss via up-regulating the P38/HIF-1α pathway on the brain of APP/PS1 transgenic mice

**DOI:** 10.3389/fnagi.2015.00104

**Published:** 2015-06-02

**Authors:** Chuang Guo, Yu-Xin Zhang, Tao Wang, Man-Li Zhong, Zhao-Hui Yang, Li-Juan Hao, Rui Chai, Shuai Zhang

**Affiliations:** ^1^College of Life and Health Sciences, Northeastern UniversityShenyang, China; ^2^Department of Anatomy, Hebei United UniversityTangshan, China

**Keywords:** Alzheimer’s disease, deferoxamine, hypoxia inducible factor, synapse, iron, transgenic mouse

## Abstract

The widely recognized neuroprotective effect of iron chelators is contributed by their ability to prevent reactive oxygen species (ROS) generation via the Fenton reaction, which sequesters redox-active Fe. An additional neuroprotective mechanism of iron-chelating compounds is to regulate the transcriptional activator hypoxia-inducible factor 1α (HIF-1α). In the present study, we observed that intranasal administration of deferoxamine decreased beta-amyloid (Aβ) deposition and rescued synapse loss in the brain of Aβ precursor protein and presenilin-1 (APP/PS1) double transgenic mice. We found that deferoxamine (DFO) up-regulated HIF-1α mRNA expression and its protein level, and further induced the proteins that are encoded from HIF-1-adaptive genes, including transferrin receptor (TFR), divalent metal transporter 1 (DMT1), and brain-derived neurotrophic factor (BDNF). The effects of DFO on the induction and stabilization of HIF-1α were further confirmed *in vitro*. This was accompanied by a decrease of Fe in the CA3 region of the hippocampus. Western blotting studies revealed that DFO differentially enhanced the phosphorylation of mitogen-activated protein kinase (MAPK)/P38 kinase *in vitro* and *in vivo*. The results suggest that the DFO may up-regulate several HIF-1-dependent neuroprotective-adaptive genes in AD via activating P38/HIF-1α pathway, which may serve as important therapeutic targets to the disease.

## Introduction

Alzheimer’s disease (AD) is the most common and progressive neurodegenerative disease. There is no effective therapy for AD. One of the characteristics of the disease is the presence of beta amyloid protein aggregates and cell and synapse loss. A large body of evidence suggests that the etiology of AD is multifactorial and results from interactions among genetic, environmental, and endogenous factors (Migliore and Coppedè, 2009). Although most AD cases are sporadic without known genetic mutations, Fe overloading appears to play an important role in the cause and/or consequence of AD during the aging process. Chelation therapy has been suggested as a valuable therapeutic approach in neurological disorders (Bandyopadhyay et al., 2010).

Deferoxamine (DFO), a metal chelator commonly used for the treatment of Fe overload, has been shown to have neuroprotective effects in animal models of stroke (Hanson et al., 2009), Parkinson’s disease (Haleagrahara et al., 2013), age-induced loss of memory (de Lima et al., 2008). However, adverse effects associated with systemic administration of DFO, including systemic metal ion loss after long-term clinical application and poor penetration through the blood-brain barrier (BBB), have limited its use in this regard (Cuajungco et al., 2000). Compared to the systemic delivery for a chronic disease such as AD, nasal drug delivery system has a good permeability and can be targeted to the central nervous system (Yue et al., 2007), reducing systemic exposure with possible side effects. We recently reported that intranasal administered DFO can alter beta-amyloid (Aβ) precursor protein (APP) processing, thereby decreasing β-Site APP cleaving enzyme 1(BACE1) and presenilin-1 (PS1) protein expression and resulting in the inhibition of Aβ deposition, neuritic plaque formation, and memory deficit in APP/PS1 transgenic mice (Guo et al., 2013a). Several molecular mechanisms of action may take part in the beneficial neurotherapeutic properties of DFO. One of the key mechanisms underlying neuroprotection via Fe chelators is their antioxidant ability. Chelators prevent Fe from redox cycling, thereby suppressing hydroxyl radical formation via the Fenton reaction (Zecca et al., 2004). Another important DFO-triggered neuroprotective mechanism in AD may result from the stabilization and transactivation of hypoxia-inducible factor-1alpha (HIF-1α) pathway (Epstein et al., 2001; Jaakkola et al., 2001; Lando et al., 2002), leading to transcriptional up-regulation of a cassette of protective genes (Zaman et al., 1999; Siddiq et al., 2005). However, whether the neuroprotective effect of intranasal administered DFO on AD transgenic mouse brain is related to the action of HIF-1α signaling pathway has not been fully clarified.

HIF-1 was identified as a α/β heterodimer, that is involved in hypoxic adaptation by transcriptionally inducing the expression of HIF-1α target genes (Wang et al., 1995; Semenza, 1998). Under hypoxia, HIF-1α is stable in the cytoplasm and can dimerize with HIF-1β in the nucleus, and then mediate the adaptation of cells to hypoxia by regulating the expression of genes involved in erythropoiesis, angiogenesis, glucose transport, and glycolysis, as well as genes relevant to Fe metabolism such as transferrin and the transferrin receptor (TFR; Semenza, 1999; Lee and Andersen, 2006; Tacchini et al., 2008). Interestingly, oxygen and Fe are required for the inhibition of HIF-1α activity (Ivan et al., 2001), thus Fe chelators can mimic hypoxia and induce HIF-1α stabilization (Mecklenburgh et al., 2002). Over the last decade, many studies have shown that DFO, which also induces and stabilizes HIF-1 expression, protects cultured cells from Aβ toxicity (Schubert and Chevion, 1995; Schubert et al., 2009) and inhibits AD progression in patients (Crapper McLachlan et al., 1991). These studies provided the clues to the possible involvement of HIF-1 in neurodegenerative disorders, which is now becoming more widely accepted.

However, despite the body of data suggesting that HIF-1 may be a good therapeutic target, more evidence is needed to further clarify the involvement of the HIF-1 signaling system in the neuroprotection contributed by Fe chelators in neurodegeneration pathologies, especially *in vivo*. Thus, we hypothesize that the neuroprotection exerted by DFO is in part due to the elimination of the HIF-1α blockade. Here, we examined the effectiveness of intranasal administration of DFO for up-regulating HIF-1α levels and subsequently activating proteins that are encoded from HIF-1-adaptive genes in APP/PS1 mice and human neuroblastoma SH-SY5Y cells stably transfected with human APP bearing the Swedish mutation (APPsw). Additionally, we determined the possible signaling pathways involved in this process.

## Materials and Methods

### Transgenic Mice and Treatments

The male APP/PS1 double transgenic mice used in this study were originally obtained from Jackson Laboratory. All experimental procedures were approved by the Laboratory Animal Ethical Committee of Hebei United University.

Mice (aged 6 months) were randomly assigned to control (*n* = 8) or DFO (*n* = 8) groups. DFO (Sigma D9533, 200 mg/kg body weight, dissolved in saline) and vehicle were administered intranasally once every other day for 3 months. For intranasal administration, a 50-μl microsyringe was used to administer four 6-μl nose drops (alternating nares with 2 min between drops; 24 μl total; 5 mg DFO) to each mouse as described in previous reports (Hanson et al., 2009). At the age of 9 months, the mice were decapitated. The brains were rapidly removed and dissected into separate hemispheres on an ice-cooled board. The hippocampus and cerebral cortex were dissected from the left hemisphere and stored at −80°C for Western blot and reverse transcription (RT)-PCR analyses. The right hemisphere was immersion fixed in 4% paraformaldehyde in phosphate buffered saline at 4°C overnight and routine paraffin sections (5 μm) were prepared for morphological analysis.

### Fe Histochemistry with Perl’s Staining

Fe staining was performed on serial sections to analyze the distribution of Fe in the brains of transgenic mice using 3, 3′-diaminobenzidine-tetrahydrochloride (DAB)-enhanced staining Perl’s staining. Briefly, sections (5 μm) were routinely dewaxed, rehydrated, and treated in 0.1 M Tris-buffer saline (TBS, pH 7.4) containing 3% hydrogen peroxide (H_2_O_2_) for 10 min to reduce endogenous peroxidase activity. The sections were then immersed in Perl’s solution containing equal parts of freshly made, aqueous potassium ferrocyanide (2%) and hydrochloric acid (2%) for 30 min. After rinsing, the sections were finally intensified with 0.025% DAB plus 0.0033% H_2_O_2_ in TBS for 10 min. The process was monitored under the microscope to avoid strong background staining, and the duration of staining was the same for each animal. After several washes with distilled water, the sections were dehydrated, covered with neutral balsam and examined with a light microscope equipped with a digital camera. All slides were digitized using the optical fractionator technique. The Fe-positive areas (number of pixels) from the brain cortex and hippocampus were manually assessed using Adobe Photoshop. The results were quantified with Image-Pro Plus 6.0 software.

### Immunohistochemistry

Routine avidin-biotinylated complex (ABC) immuno-histochemical staining was performed to analyze the distribution of HIF-1α in the APP/PS1 transgenic mouse brain. Briefly, paraffin sections were dewaxed, rehydrated and treated in 0.1 M TBS containing 3% hydrogen peroxide for 10 min to reduce endogenous peroxidase activity. After washing with TBS, sections were boiled in citric acid buffer for 3 min in a microwave oven. The sections were then rinsed, treated with 5% bovine serum albumin for 30 min, and subsequently incubated overnight with rabbit anti- HIF-1α (1:200; Novus NB100-479) at 4°C. After rinsing, sections were incubated with biotinylated goat anti-rabbit IgG (1:200) for 1 h, followed by streptavidin peroxidase incubation for 1 h at room temperature. After rinsing, the sections were stained with 0.025% DAB for 3 min. The stained sections were dehydrated, cleared, covered with neutral balsam, and examined under a light microscope equipped with a digital camera. Control sections were treated with identical solutions but without primary antibody followed by all subsequent incubations as described above.

### Cell Culture, Drug Treatment

The APPsw cells were maintained in Dulbecco’s modified Eagle’s medium and Ham’s F-12 nutrient mixture supplemented with 10% heat-decomplemented fetal calf serum, 100 IU/mL penicillin, 100 μg/mL streptomycin and 200 μg/mL G418 at 37°C in humidified 5% CO_2_ air. Serum-free medium was added for 2 h when cells reached nearly 70–80% confluence before drug treatments. Cells were treated with FeSO_4_ (100 μM) or SB203580 (10 μM, a P38 MAPK inhibitor) for 2 h and then treated with DFO (100 μM). To ensure that the potential effects of Fe and DFO on HIF-1α protein expression were not due to treatment-induced cell death, the concentrations of Fe and DFO selected were based on previously described studies (Guo et al., 2013a). After 24 h, the cells were collected for Western blot analysis.

### Western Blotting

For Western blots, the APP/PS1 transgenic mouse brain tissues and culture cells were homogenized in ice-cold lysis buffer containing a mixture of protease inhibitors. After homogenates were centrifuged at 12,000 rpm for 30 min at 4°C, supernatants were collected and the total protein levels were measured using a UV 1700 PharmaSpec ultraviolet spectrophotometer. Equal amounts proteins were resolved in 10% sodium dodecyl sulfate polyacrylamide gels and blotted on polyvinylidene fluoride membranes (Millipore, CA, USA). After blocking in 5% non-fat milk for 1 h, the membranes were incubated sequentially overnight at 4°C with the following primary antibodies: rabbit anti-APP695 (1:4000, Chemicon), rabbit anti- DIVALENT METAL TRANSPORTER 1 (DMT1)-IRE (1:3000, Alpha diagnostic), rabbit anti- DMT1-noIRE (1:3000, Alpha diagnostic), rabbit anti- HIF-1α (1:1000, Novus), rabbit anti-p-JNK (1:1000, Cell Signaling Tech), rabbit anti-JNK (1:1000, Cell Signaling Tech), rabbit anti-p-ERK (1:1000, Cell Signaling Tech), rabbit anti-ERK (1:1000, Cell Signaling Tech); rabbit anti-p-P38 (1:500, Santa Cruz), rabbit anti-P38 (1:500, Santa Cruz); rabbit anti-synaptophysin (SYP; 1:1000; Sigma-Aldrich); mouse anti-TFR (1:1000, Invitrogen); mouse anti- brain-derived neurotrophic factor (BDNF; 1:500, Chemicon), and mouse anti-GAPDH (1:10000, Kang Chen, KC-5G5). After washing, the membranes were incubated with horseradish peroxidase (HRP)-labeled secondary antibodies (1:5000; Santa Cruz) for 1 h. Blots were visualized using Super Signal West Pico Chemiluminescent Substrate (Pierce Biotechnology, Rockford, IL, USA) and Chem Doc XRS with Quantity One software (BioRad, USA). The band intensities were quantified using Image-pro Plus 6.0 analysis software.

### RT-PCR

Total RNA was extracted from APP/PS1 transgenic mouse brain tissue samples using Trizol (Invitrogen). After quantification by UV-spectroscopy at 260 nm, total RNA of each sample was first transcribed to cDNA using EasyScriptTM Two-Step RT-PCR SuperMix (TransGen Biotech, AE401). PCR amplification was performed with reagents from TransGen Biotech. The cDNA solution was amplified with sequences of the following primers: HIF-1α, forward: TGGTATTATTCAGCACGAC, reverse: GAGACATTGCCAGGTTTAT; glyceraldehyde 3-phosphate dehydrogenase (GAPDH), forward: ACGGATTTGGTCGTATTGGG, reverse: CGCTCCTGGAAGATGGTGAT. The PCR conditions were: HIF-1α, 30 cycles of 94°C for 45 s, 50°C for 45 s and 72°C for 45 s; GAPDH, 30 cycles of 95°C for 45 s, 58°C for 45 s, and 72°C for 60 s. The PCR products were normalized with reference to GAPDH mRNA. The results were quantified with ChemDoc XRS Quantity One software.

### Immunohistochemistry and Confocal Laser Scanning Microscopy

Cultured cells were seeded to sections. After treatment, cell culture medium was carefully removed. After rinsing in PBS, the cells were fixed in a freshly prepared solution of 4% paraformaldehyde for 30 min. For immunofluorescent double staining, sections or cell cultures were preincubated with normal donkey serum (1:20; Jackson ImmunoResearch Laboratory) for 30 min and then incubated overnight with either mouse anti-Aβ (1:500; Sigma-Aldrich) and rabbit anti-SYP (1:200; Sigma-Aldrich), or mouse anti-Aβ and rabbit anti-HIF-1α (1:200). After three washing with PBS, the immunoreactivity was probed using Texas-Red or fluorescein isothiocyanate (FITC) Green conjugated secondary antibodies (1:200; Jackson ImmunoResearch Laboratory) for 2 h at room temperature. After rinsing, the sections and cells were stained with DAPI for 5 min. The images were observed by using a confocal laser scanning microscope (SP2, Leica). No fluorescence is expected to be detected when the primary antibody is omitted.

### Statistical Analysis

All data are expressed as the mean ± standard error (S.E.). Differences between means were evaluated with Student’s *t*-test, and two-way ANOVA with *Post hoc* Fisher’s Protected Least Significant Difference (PLSD) for cultured cells. All data were analyzed using SPSS 13.0 software, and statistical significance was assumed if *p* < 0.05.

## Results

### Intranasal DFO Treatment Modulated the Distribution of Fe in the Brains of APP/PS1 Mice

**Figure 1:**
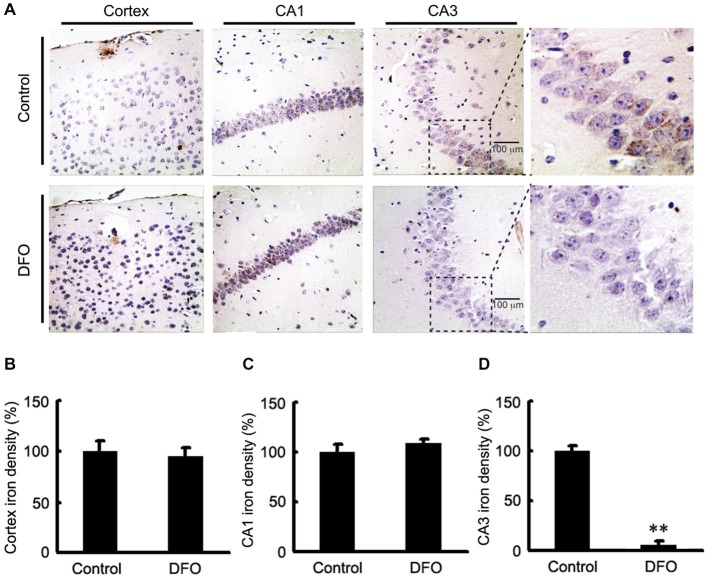
**Fe accumulation in the cortex and hippocampus of APP/PS1 mice. (A)** APP/PS1 transgenic mice were treated with control or DFO (200 mg/kg) for 3 months before sacrifice. The right hemisphere of the brains were quickly dissected, fixed in 4% paraformaldehyde in phosphate buffered saline at 4°C overnight, embedded in paraffin, cut into sections (5 μm) and stained with modified Perl’s-DAB to reveal the distribution patterns of Fe ions in the cortex and hippocampus. Fe deposits can be observed as brown granules. In the cortex and hippocampus, there are many Fe-positive cells, most of which are neurons with cytoplasmic Fe deposits, especially CA1 area of hippocampus. Importantly, Fe ions were only detected in the CA3 area of the hippocampus untreated mice. **(B–D)** Bar graphs illustrating the semi-quantitative Fe ions densitometry measurements as percentages of the intact side. Scale bar = 100 μm. These images are representative of 6 independent experiments, all revealing similar results. **P < 0.01 vs. control group.

We previously showed DFO did not attenuate the total content of Fe in brains tissues of APP/PS1 mice when it was administred (Guo et al., 2013a). In this study, brain sections from control and APP/PS1 mice that were intranasally administered DFO were subjected to Perl’s-DAB analysis as a relative measure of Fe content within plaques and tissue. In general, Fe-positive plaques were distributed throughout the cortex and hippocampus in all examined animals (Figure 1), as previously described (Moreira et al., 2010). Fe deposits were observed as brown granules. In the cortex and hippocampus, many Fe-positive cells were detected, most of which were neurons with cytoplasmic Fe deposits, especially in the CA1 area of the hippocampus (Figure 1A). Importantly, Fe ions were only detected in the CA3 area of the hippocampus in untreated mice (Figure 1D), while no significant changes were observed in the cortex and the CA1 area of the hippocampus (Figures 1B,C). Thus, we can infer that mediating the redistribution of Fe rather than chelated Fe is probably responsible for inhibition of APP/Aβ pathology in the brains of DFO-treated APP/PS1 transgenic mice.

### Intranasal DFO Treatment Reduced Synapse Loss in APP/PS1 Mouse Brains

**Figure 2:**
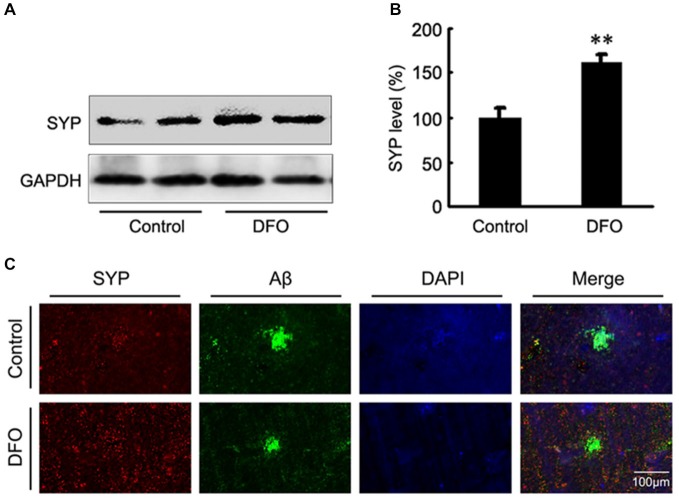
**Intranasal DFO treatment reduced synapse loss in APP/PS1 mouse brain. (A,B)** Western blot analysis showed that DFO treatment significantly increased the expression level of SYP compared with control group. GAPDH was used as a loading control. **(C)** Immunofluorescence labeling and confocal microscopy analysis showing the distribution and expression of SYP (red) and Aβ (green) in brain sections of APP/PS1 mice. The positive area of SYP staining was significantly increased following a decreased immunoreactive area of Aβ plaque in the brain of DFO-treated APP/PS1 mice, compared with controls. Scale bar = 100 μm. Data represent the mean ± S.E. of 6 independent experiments. **P < 0.01 vs. control group.

Previous studies have proposed that intranasal DFO administration reduced the expression and phosphorylation of APP protein by shifting the processing of APP to the non-amyloidogenic pathway. This reduction was accompanied by an attenuation of both the Aβ burden and tau phosphorylation, and memory retention was significantly promoted in APP/PS1 mice (Guo et al., 2013a,b). In the present study, we then assessed the effects of DFO on synapse lesion. As shown in Figures 2A,B, Western blot analysis showed that DFO treatment significantly increased the expression level of SYP compared with control group. As further evidence of that, the positive area of SYP staining was increased accompanied with a decreased immunoreactive area of Aβ plaque in the brain of DFO-treated APP/PS1 mice, compared with controls (Figure 2C). These results suggest that intranasal DFO treatment reduce synapse loss, and has a neuroprotective effect on the brain of APP/PS1 transgenic mice.

### Effect of DFO on the Expression of HIF-1α and Proteins that are Encoded from HIF-1-Adaptive Genes in the Brains of APP/PS1 mice

**Figure 3:**
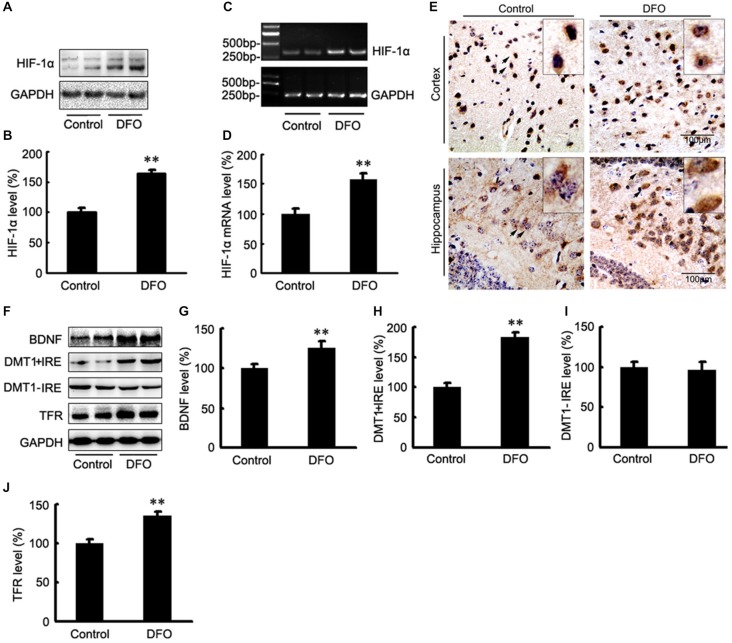
**DFO regulates the expression of HIF-1α and HIF-1-related protein in the brains of APP/PS1 mice. (A)** Western blots showing the expression levels of HIF-1α protein in control and DFO-treated transgenic mice brains. **(B)** Immunoblot showed that DFO treatment significantly increased HIF-1α protein levels, compared with controls. **(C)** The expression levels of HIF-1α mRNA were detected by RT-PCR in the brains of APP/PS1 transgenic mice treated with DFO. **(D)** RT-PCR analysis showed that DFO treatment significantly increased the HIF-1α mRNA levels in the transgenic mouse brain. GAPDH served as the internal control. **(E)** Immunohistochemically stains showed the distribution of HIF-1α in the cortical and hippocampus sections of APP/PS1 mouse brain (Scale bar = 100 μm). **(F)** Western blots showing the expression levels of brain-derived neurotrophic factor (BDNF), DMT1, and TFR proteins in APP/PS1 transgenic mice brains 3 months after DFO administration. GAPDH was used as a loading control. **(G–J)** DFO treatment led to a marked increase in the BDNF, DMT1 + IRE, and TFR protein levels in the brains of transgenic mice compared with the control. There was no significant change in the expression levels of DMT1-IRE protein between the groups, compared with the control. Data represent the mean ± S.E. of 6 independent experiments. **P < 0.01 vs. control group.

The potential therapeutic effect of a Fe-chelating compound may involve the activation of Fe-dependent prolyl hydroxylases, which regulate HIF stability, leading to transcriptional up-regulation of a cassette of target genes. In turn, the up-regulation of these genes could contribute to neuroprotection (Wang et al., 1995; Zaman et al., 1999). To investigate the role of DFO in HIF-1α gene regulation, we first determined the mRNA levels of HIF-1α in APP/PS1 mice brains by RT-PCR analyses. As shown in Figure 3, the expression levels of both HIF-1α protein (Figures 3A,B) and mRNA (Figures 3C,D) were significantly increased after intranasal DFO treatment, compared with the control mice (*p* < 0.01), indicating that the drugs not only stabilized HIF-1α protein but influenced its transcriptional regulation. Immunohistochemical staining strengthened the biochemical results and further revealed that intranasal DFO treatment caused not only accumulation but also nuclear translocation of HIF-1α (Figure 3E). The HIF-1α expressed in the control was almost exclusively distributed in the cytoplasm of the neurons and rarely found in the nucleus. In contrast, cells of the DFO-treated group in the cortex and dentate gyrus of the hippocampus expressed high levels of HIF-1α in both the cytoplasm and nucleus (Figure 3E).

Altogether, these results suggest that DFO has a positive effect on HIF-1α gene and protein expression in the APP/PS1 mouse brain.

To determine whether DFO-induced HIF-1α was transcriptionally active, we further examined the effects of DFO on the expression levels of selected downstream target proteins of HIF-1α that might be involved in mediating Fe metabolism and neuroprotection. Quantitative analyses of Western blots revealed that treatment of APP/PS1 mice with DFO significantly up-regulated the expression of BDNF, DMT1 + IRE, and TFR proteins (*p* < 0.01; Figures 3F–H,J), whereas there were no significant changes in the expression levels of DMT1-IRE protein between the DFO-treated and control groups (*p* > 0.05; Figures 3F,I). These results are consistent with previous reports demonstrating that Fe deficiency results in HIF-1α stabilization and the up-regulation of several HIF-dependent adaptive genes, thereby coordinating the response to hypoxia conditions (Wang et al., 1995; Soucek et al., 2003).

### Effects of DFO on HIF-1α Regulatory Pathways

**Figure 4:**
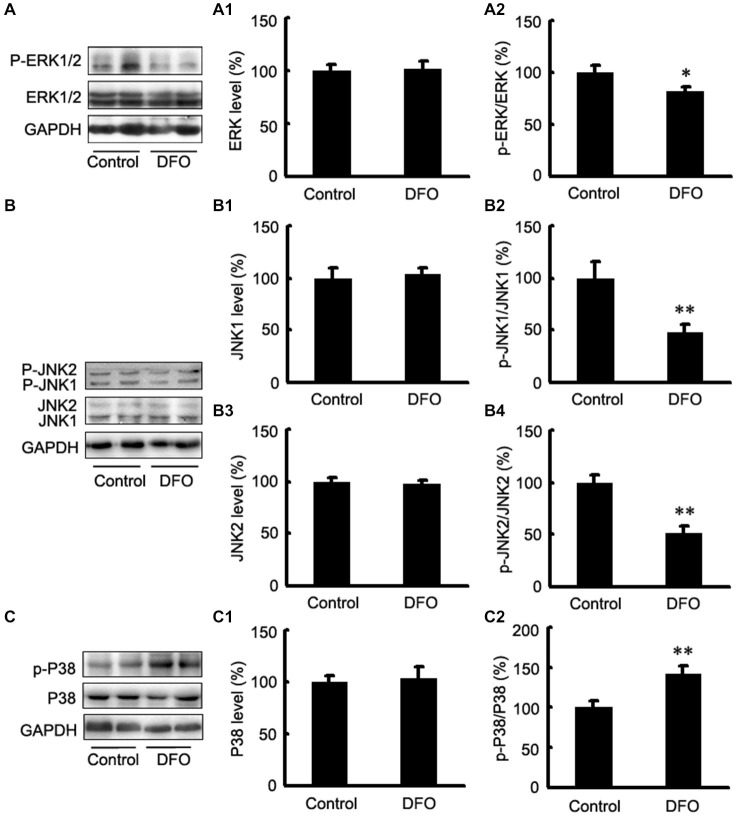
**Expression levels of ERK, JNK, and P38 in the brains of APP/PS1 mice 3 months after DFO treatment. (A)** Western blots showing the protein levels of ERK1/2 in the brains of transgenic mice. **(A1,A2)** There were no significant differences in total ERK1/2 levels between the brains of control and DFO-treated. However, the ratio of p-ERK1/2 to ERK1/2 was significantly decreased after DFO treatment. **(B)** Immunoblot analyses showing the expression levels of JNK1/2 in the brains of transgenic mice. **(B1–B4)** A marked reduction in the ratio of p-JNK1/2 to JNK1/2 was detected in the brains of DFO-treated mice, whereas there were no significant differences in total JNK1/2 levels between the brains of control and DFO-treated mice. **(C)** Western blots showing the protein levels of P38 in the brains of transgenic mice. **(C1,C2)** There were no significant differences in total P38 levels between the brains of control and DFO-treated mice. However, the ratio of p-P38 to P38 was markedly increased after DFO treatment. GAPDH was used as an internal control. Data represent the mean ± S.E. of 6 independent experiments. *p < 0.05, **p < 0.01 vs. control group.

It has been reported that the mitogen-activated protein kinase (MAPK) signaling pathway and phosphatidylinositol-3-kinase (PI3K)/AKT signaling play important roles in regulating HIF-1α expression (Blancher et al., 2001; Jiang et al., 2001). Here, we investigated the activation of the three MAPKs by Western blot analysis using phosphorylation-specific MAPK antibodies in DFO-treated APP/PS1 mice (Figure 4). Treatment with DFO induced a significant decrease in phosphorylated ERK and JNK levels (*p* < 0.05, Figure 4A; *p* < 0.01, Figure 4B). However, treatment with DFO significantly increased the levels of p-P38 MAPK (*p* < 0.01, Figure 4C) without affecting the total amounts of ERK, JNK, and P38 MAPK proteins (Figure 4). These results suggest that DFO-induced HIF-1α protein accumulation was, at least in part, dependent on P38MAPK-mediated signaling pathway.

### DFO Inhibits Aβ Generation Involved in HIF-1α Stabilization and the Expression of Proteins that are Encoded Form HIF-1-Adaptive Genes in APPsw Cells

**Figure 5:**
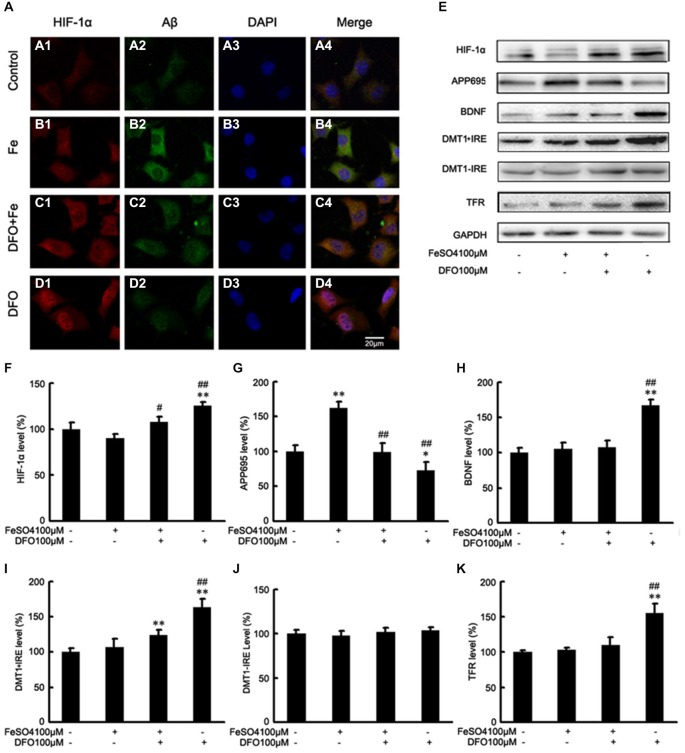
**DFO inhibits Aβ generation involved in HIF-1α stabilization and the expression of proteins that are encoded form HIF-1-adaptive genes in APPsw cells. (A)** Immunofluorescence labeling and confocal microscopy analysis showing the distribution and expression of HIF-1α **(A1–D1)** and Aβ **(A2–D2)** in APPsw cells. DFO treatment significantly increased the HIF-1α immunofluorescence **(D1)** and reduced the Aβ immunofluorescence **(D2)** in APPsw cells compared with the control **(A1,A2). (A3–D3)** DAPI was used to detect the nucleus (blue). **(A4–D4)** Merged images from the double channels indicated that DFO has an inhibitory effect on Aβ formation is involved in HIF-1α stabilization in APPsw cells. Fe treatment (100 μM) decreased the levels of HIF-1α **(B1)** and increased the Aβ immunofluorescence **(B2)**. The additional of DFO treatment reversed the reduction of HIF-1α **(C1)** and the increase of Aβ **(C2)** in cultures pretreated with 100 μM Fe. The images are a representative of three independent experiments. Scale bar = 20 μm. **(E)** Western blot analysis was performed to determine the expression levels of HIF-1α, APP695, BDNF, DMT1, and TFR; GAPDH served as the internal control. **(F)** There were no significant changes in HIF-1α levels in the Fe- or Fe + DFO-treated groups compared with the controls. HIF-1α was markedly increased after DFO treatment. In the Fe + DFO group, the level of HIF-1α was significantly increased compared with that in the Fe treatment group. **(G)** Fe treatment significantly increased the levels of APP695. In the Fe + DFO group, the levels of APP695 were significantly decreased compared with the Fe treatment group. DFO treatment significantly reduced the expression level of APP695 compared with their levels in the controls. **(H–K)** There were no significant changes in BDNF, DMT1 + IRE, and TFR levels in the Fe-treated groups, whereas the level of DMT1 + IRE was significantly increased in the Fe + DFO compared with the controls. The level of BDNF, DMT1 + IRE, and TFR was significantly increased in the DFO group, compared with the controls. No differences in the expression levels of DMT1-IRE were found between the controls and any of the treatments. Data represent the mean ± S.E. (*n* = 3). *p < 0.05, **p < 0.01 compared with the control group; ^#^*p* < 0.05, ^##^*p* < 0.01 compared with the Fe treatment group (two-way ANOVA with *Post hoc* Fisher’s PLSD).

To further investigate the mechanism underlying DFO-mediated up-regulation of HIF-1α and the neuroprotective effect, we examined the effects of DFO against ferrous Fe-induced toxicity using Western blot analysis *in vitro*. To avoid the influence of cell death in the following experiments, we have previously explored the optimal concentrations of Fe and DFO, as following: 100 μM ferrous sulfate and/or 100 μM DFO (Guo et al., 2013a). As shown in Figure 5, there were no significant differences at the protein levels of HIF-1α, BDNF, DMT1 + IRE, and TFR between the Fe group and the control cells. However, in DFO-treated cells, chelation of Fe with 100 μM DFO significantly increased the expression levels of HIF-1α, BDNF, DMT1 + IRE, TFR proteins, respectively, compared with the control cells (*F* = 7.014; *F* = 18.327; *F* = 15.216; *F* = 8.835; *p* < 0.01; Figures 5E,F,H,I,K). Notably, HIF-1α and DMT1 proteins in the Fe + DFO-treated cells were significantly increased compared with their levels in control cells (*p* < 0.01; Figures 5E,F,I). In addition, no differences in the expression levels of DMT1-IRE were found between the controls and any of the treatments (*F* = 1.965; *p* > 0.05; Figures 5E,J).

Next, we also performed Western blot analysis to determine the protein levels of APP695 in cells treated with DFO and/or Fe. Immunoblotting revealed that, compared with controls, treatment with Fe resulted in significantly increased levels of APP695 protein (*F* = 11.590; *p* < 0.01, Figures 5E,G). In contrast, the expression levels of APP695 in the DFO group were significantly decreased compared with controls (*p* < 0.01; Figures 5E,G). Moreover, these increases were abolished when the cultures were treated with Fe followed by DFO (*F* = 20.819; *p* < 0.01; Figures 5E,G). Double labeling of Aβ (Figures 5A2–D2) and HIF-1α (Figures 5A1–D1) showed that treatment with Fe significantly increased the Aβ immunofluorescence in APPsw cells compared with the control (Figures 5A1,A2,B1,B2). DFO treatment decreased the immunofluorescence intensity of Aβ but increased HIF-1α immunoreactive intensity (Figures 5C1,C2) when the cells were treated with Fe and DFO. Levels of HIF-1α were detected in both the cytoplasm and the nucleus in the DFO-treated cells. These results suggest that DFO has an inhibitory effect on APP695 protein expression and Aβ formation is involved in HIF-1α stabilization in APPsw cells.

Collectively, these results further suggest that DFO significantly induces HIF-1α accumulation, and promotes nuclear translocation, and then suppresses the Aβ generation.

### DFO up-Regulate HIF-1α Protein by the Activation of P38 MAPK in APPsw Cells

**Figure 6:**
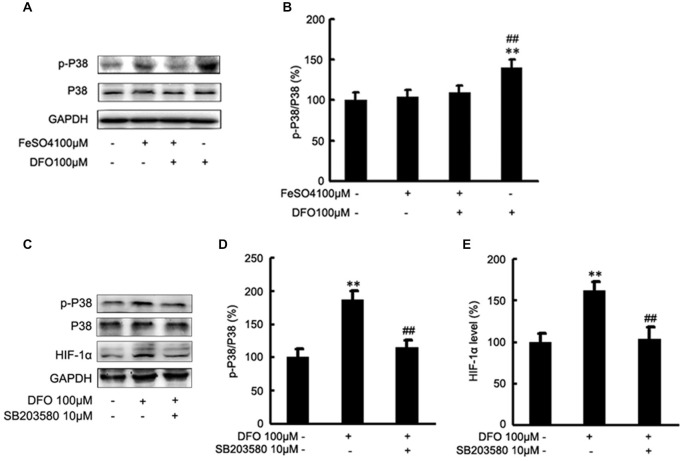
**Roles of P38 mitogen-activated protein kinase (MAPK) in the DFO-mediated up-regulate of HIF-1α protein in APPsw cells. (A)** APPsw were exposed to 100 μM Fe, 100 μM DFO, or both combined for 24 h. Whole cell lysates were prepared and subjected to analysis for P38 or GAPDH protein. **(B)** Western blot analysis revealed that strong phosphorylation of P38 MAPK was detected in the DFO-treated cells, whereas no significant changes were observed in cells treated Fe alone compared with control. There were not statistical changes in the levels of P38 MAPK among the groups. The data represent the mean ± S.E. of three independent experiments. *p < 0.05, **p < 0.01 compared with the control; ^#^*p* < 0.05,^ ##^*p* < 0.01 with respect to the Fe treatment group. **(C–E)** APPsw cells were pretreated without or with SB203580 (10 μM) for 2 h and then treated without or with DFO for additional 22 h in the absence or presence of the inhibitor. Immunoblotting showed that DFO treatment significantly increased the levels of p-P38 MAPK compared with the control group, whereas the increase was significantly ameliorated by SB203580. Furthermore, the DFO-mediated up-regulation of HIF-1α protein was greatly inhibited by SB203580. The data represent the mean ± S.E. (*n* = 3). **p < 0.01 compared with the control; ^##^*p* < 0.01 compared to the values of DFO treatment group (two-way ANOVA with *Post hoc* Fisher’s PLSD).

To test the hypothesis that DFO may up-regulate the HIF-1α signaling cascades by P38 MAPK activation in APPsw cells, we further investigated the effect of DFO on the activation of intracellular signaling proteins in APPsw cells. As shown in Figure 6, strong phosphorylation of P38 MAPK was detected in the DFO-treated cells (*F* = 5.321; *p* < 0.01; Figures 6A,B), whereas no significant changes were observed in cells treated with Fe alone compared with the control group. Interestingly, the total P38 MAPK protein level, were not changed among the groups (*p* > 0.05; Figures 6A,B).

Then, inhibition studies were carried out to investigate the effect of signaling proteins in the DFO-mediated up-regulation of HIF-1α. For this, APPsw cells were treated without or with DFO in the absence or presence of SB203580. Immunoblotting showed that DFO treatment at 24 h significantly increased the phosphorylation of P38 MAPK compared with the control group, whereas the increase was significantly ameliorated by SB203580 (*p* < 0.01; Figures 6C,D). Profoundly, as shown in Figures 6C,E, the DFO-mediated up-regulation of HIF-1α protein in APPsw cells was greatly inhibited followed by the pretreatment with SB203580.

Taken together, these data suggest that activities of P38 MAPK, specifically the phosphorylation of P38 MAPK, contribute to the increased expression and activity of HIF-1α protein in APPsw cells upon DFO treatment.

## Discussion

DFO is a Fe chelator that has been shown to prevent AD in a clinical trial and a range of disease models (Crapper McLachlan et al., 1991; Savory et al., 1998; Morse et al., 2004; Fine et al., 2012; Guo et al., 2013a,b). Our previous studies have demonstrated that intranasal DFO treatment reverses Fe-induced memory defects and inhibits amyloidogenic APP processing and tau hyperphosphorylation in APP/PS1 mice (Guo et al., 2013a,b). Despite these important advances, the mechanisms of DFO-induced neuroprotection are not completely understood. This present study was designed to address the mechanism of intranasal DFO-induced hypoxia signaling to further our understanding of the therapeutic action of this agent in AD pathogenesis. DFO treatment decreases the Aβ deposition and reduces the synapse loss. More importantly, we extend previous observations and demonstrate that DFO-induced HIF-1α stabilization regulates Fe homeostasis and is a target for neuroprotection. These neuroprotective mechanisms include functional activation of HIF-1 signaling and regulation of wide range of HIF-1-related adaptive proteins, such as DMT1 + IRE, TFR and BDNF. Consequently, we have now identified important roles of the P38 MAPKs in the intranasal DFO-induced HIF-1α upregulation in APP/PS1 mice and APPsw cells.

Fe chelators, which can possess Fe chelating ability for removal of excess Fe in the brain, have the potential for clinical use in neurological disorders, therefore, they have been suggested as a potential noninvasive strategy for the treatment of AD (Hanson et al., 2009; Fine et al., 2012; Guo et al., 2013a). In this study, we found that Fe-containing cells in the cortex and the CA1 area of hippocampus did not significantly differ in amounts between the DFO-treated and the control mice, whereas the levels of Fe in the Fe-positive cells in DFO-treated mice were significantly reduced in the CA3 area of the hippocampus. Therefore, we presume that the redistribution of Fe, rather than the removal of Fe from the system, is most likely responsible for the neuroprotective functions of DFO.

Recently, alternative mechanisms involving chelator exposure were proposed for example, Fe chelator exposure can induce the cellular stress response by activating adaptive signaling proteins such as HIF-1α. This response triggers gene expression shifts that increase the ability of the cell to tolerate elevated levels of oxidative stress, and more importantly, cellular Fe dyshomeostasis (Lee and Andersen, 2006). Indeed, previous *in vitro* studies demonstrated that DFO treatment may cause a decrease in mitochondrial reactive oxygen species (ROS) levels in primary neurons, which then act as signaling molecules for HIF-1 activation (Bianchi et al., 1999; Zaman et al., 1999; Honda et al., 2005; Siddiq et al., 2005); the induction of HIF-1α activation and gene signaling associated with Fe homeostasis and metabolism (Lee et al., 1997; Rolfs et al., 1997). The current study demonstrated that intranasal DFO administration induced the mRNA expression of HIF-1α and increased protein levels of HIF-1α in APP/PS1 mouse brains. Subsequently, DFO significantly up-regulated the expression levels of several adaptive HIF-1-related genes, such as BDNF and the Fe homeostasis/metabolism protein DMT1 + IRE, TFR, implying an additional neuroprotective mechanism of DFO: induction of HIF-1α and regulation of Fe metabolism *in vivo* (Semenza, 1998; Bergeron et al., 1999). These results are further supported by the findings that increased levels of HIF-1α were detected in both the cytosol and the nucleus of cortical and hippocampal neurons in the DFO-treated group. Additionally, studies with SH-SY5Y cells *in vitro* demonstrated that epigallocatechin-3-gallate (EGCG) was to confer neurorescue effects and significantly up-regulate the expression of HIF-1 and its target gene TFR, the latter is associated with Fe homeostasis (Reznichenko et al., 2006; Weinreb et al., 2008). Interestingly, our study also demonstrated that DFO elevates BDNF protein levels, which promotes neuronal survival and differentiation (Thoenen, 1995, 2000; Schinder and Poo, 2000). It also was reported that M30 increases the expression levels of BDNF transcripts (Avramovich-Tirosh et al., 2010), thereby inhibiting hypoxia-induced apoptosis (Ruscher et al., 2002). To further corroborate the role of HIF-1α in the pathogenesis of AD, we embarked in a series of *in vitro* experiments. In accordance, our *in vitro* findings demonstrated that DFO up-regulated expression of HIF-1α and several HIF-1α target proteins in APPsw cells, which was accompanied by a reduction in APP695 protein expression and Aβ generation. This can be viewed that the regulation of HIF-1α expression and its related targets may constitute an additional pathway underlying the neuroprotective effect of DFO.

Notably, the modulation of the HIF pathway appears to be complex. Some proteins have been shown to stimulate HIF-1α transactivation or synthesis by activation of the MAPK or the PI3K signaling pathways (Zelzer et al., 1998; Li et al., 2004; Lonati et al., 2014). To elucidate the molecular mechanism for the DFO induced HIF-1α stabilization and activation, we evaluated the total and activated forms of ERK1/2, JNK1/2, and P38 because they have been also implicated in involving APP processing with increased Aβ deposition (Atkins et al., 1998; Savage et al., 2002; Kim et al., 2006; Cho et al., 2007; Lee et al., 2009). Here, we showed that the phosphorylation of ERK1/2 and JNK was attenuated in the DFO-treated group *in vivo*. Our data are in agreement with a very recent study describing that green tea catechin, an iron chelator, may decrease in behavioral impairment, Aβ-42 production, and activation of ERK and JNK MAPK in NSE/hAPP-C105 transgenic mice (Lim et al., 2013). Interestingly, we observed that under our experimental conditions, DFO enhanced P38 MAPK phosphorylation in cultured APPsw cells and brains of treated APP/PS1 mice. It has been reported that different MAPKs are activated by different stimuli and target different downstream molecules; therefore, they each perform different functions (Schaeffer and Weber, 1999; Roux and Blenis, 2004). Therefore, these alterations in the expression of MAPK family members induced by DFO treatment provide vital information about the mechanism of DFO action during AD treatment. Further, we observed *in vitro* that inhibitors of P38 block HIF-1α upregulation by DFO treatment. These results, and the demonstration of P38 MAPK elevation in both human APPsw cells and AD mouse brains, suggest that P38 MAPK is a central mediator of hypoxia-induced neuroprotection in AD. The notion that DFO might regulate HIF-1α via phosphorylation of MAPK/P38 signaling pathway will be addressed in greater depth in future studies.

Taken together, the present results suggest that the neuroprotective effects of DFO may be correlated, at least in part, with the ability of this Fe chelator to activate the transcriptional activator HIF-1α and up-regulate HIF-1α-mediated protective genes, especially these targets involving in Fe metabolism. Our data further indicate that HIF-1 may prevent AD occurrence, postpone AD progression, and ameliorate AD symptoms by regulating the expression of its target genes. Thus, Fe chelators, and specifically intranasal administration of DFO, might be beneficial for their potent efficacy in the prevention and treatment of neurodegenerative diseases such as AD.

## Conflict of Interest Statement

The authors declare that the research was conducted in the absence of any commercial or financial relationships that could be construed as a potential conflict of interest.

## Abbreviations

Aβ: amyloid-β
AD: Alzheimer’s disease
APP: amyloid precursor protein
APPsw: amyloid precursor protein Swedish mutant
BACE1: β-Site APP cleaving enzyme 1
BDNF: brain-derived neurotrophic factor
DFO: deferoxamine
DMT1: divalent metal transporter 1
GAPDH: glyceraldehyde 3-phosphate dehydrogenase
GSK-3β: glycogen synthase kinase-3β
HIF: hypoxia inducible factor
HRE: hypoxia-response element
HRP: horseradish peroxidase
IHC: immunohistochemistry
MAPK: mitogen-activated protein kinase
PS1: presenilin 1
TBS: Tris-buffered saline
TFR: transferrin receptor.
